# Enhancing the Comprehensive Integration of General Medicine Education in Rural Japan: A Thematic Analysis

**DOI:** 10.7759/cureus.50874

**Published:** 2023-12-20

**Authors:** Ryuichi Ohta, Chiaki Sano

**Affiliations:** 1 Community Care, Unnan City Hospital, Unnan, JPN; 2 Community Medicine Management, Shimane University Faculty of Medicine, Izumo, JPN

**Keywords:** community healthcare perspective, interprofessional collaboration, rural medical education, educational settings, medical trainees, clinical transition, thematic analysis, lateral integrations, curriculum revision, general medicine education

## Abstract

Introduction

The dynamism inherent in general medicine, particularly since its recognition as a distinct specialty in 2019, necessitates constant revision and refinement of the curriculum. As general medicine programs proliferate throughout Japan, understanding the revision processes, especially concerning the pivotal concept of lateral integrations, becomes critical. Lateral integrations, which pertain to the interconnectedness between learning contents and contexts, ensure a cohesive learning experience for medical students. In this study, we sought to explore the intricacies and experiences of revising these integrations within the general medicine curriculum.

Methods

A qualitative thematic analysis rooted in relativist ontology and constructivist epistemology was conducted. The research was carried out at the Unnan City Hospital, Shimane Prefecture, focusing on trainees transitioning between diverse medical settings. Semi-structured interviews were employed to gauge perceptions regarding these transitions, and thematic analysis was used to interpret the data. Reflexivity was ensured by the diverse expertise of the research team, with rigorous discussions to mitigate biases.

Results

The following four themes emerged from the analysis: (1) confusion due to the transition from acute to chronic clinical settings, with trainees feeling overwhelmed and resistant to focus solely on chronic care; (2) monotony due to the loss of some clinical experiences, indicating challenges in maintaining motivation after transitioning to clinics; (3) disconnection between learning contexts, where participants desired stronger links to their primary training hospitals; and (4) anxiety as community leaders, highlighting the need for instilling leadership skills and a deeper understanding of diverse community healthcare professions.

Conclusion

This study shed light on the tangible challenges faced by general medicine trainees during transitions between different learning environments. These insights are valuable for educators in refining curriculum structures, ensuring smooth transitions, and enhancing lateral integrations. Addressing these challenges will bolster the quality and relevance of general medicine education in Japan, fostering the creation of adaptable, well-rounded physicians who are attuned to the multifaceted needs of their communities.

## Introduction

In medical education, continual revision and refinement of curricula is paramount to ensure they remain reflective of the current needs and best practices [1]. This is especially crucial in a field as dynamic and multifaceted as general medicine [2]. Recognized as a distinct specialty in 2019, the field of general medicine education has witnessed the establishment of diverse programs across Japan, aiming to cultivate well-rounded physicians capable of handling the complexities of patient care [3,4].

Professional teachers committed to enhancing the structure and content of general medicine curricula are at the heart of this ongoing development. Their collaborative efforts with various stakeholders, including medical teachers and learners, are fundamental to fostering an environment conducive to progressive learning and application [5,6]. In this context, constant reevaluation of educational methods emerges as an essential mechanism for improving learning qualities and addressing the medical community's and patients' ever-evolving needs [7]. Developing multiple-year curricula for general medicine education necessitates incorporating various essential elements. Among these, the concept of lateral integrations is pivotal [8]. Horizontal integration refers to the interconnectedness and coherence between different learning contents and contexts within the curriculum, ensuring a comprehensive and cohesive learning experience for medical students [9]. 

In this study, we aim to explain the processes involved in revising the lateral integration between learning contents and contexts within the curriculum to clarify the difficulties and challenges that medical learners encounter in the lateral integration of general medicine education. We aimed to uncover perceptions and experiences by employing a qualitative research approach. The insights derived from this investigation will provide valuable perspectives on the effectiveness and areas for improvement of the existing integrative structures within the general medicine education curriculum. Ultimately, this research seeks to contribute to the ongoing efforts to enhance the quality and relevance of general medicine education in Japan, paving the way for the development of competent and adaptable physicians equipped to meet society's diverse healthcare needs.

## Materials and methods

Study design

This study used thematic analysis based on ontology and epistemology for research. General medicine trainees in Japan have diverse backgrounds, prior experience, and an interest in medicine. They train in the general medicine curriculum from different perspectives, which can make their learning contextual and self-directed. We examined from a prism of relativist ontology and constructivist epistemology to investigate their perceptions of the general medicine curriculum for learning in terms of context. Thus, the thematic analysis was based on a qualitative framework [10].

Setting

The study was conducted at the Unnan City Hospital in rural southeastern Shimane, Japan. The hospital has 281 care beds: 160 for acute care, 43 for comprehensive care, 30 for rehabilitation, and 48 for chronic care. It provides general medicine training to medical residents. Under this curriculum, medical residents experience multiple clinical scenarios in treating their patients: inpatient, outpatient, home, and community care. The General Medicine department mainly manages its educational curriculum. The department promotes interprofessional collaboration with dentists, pharmacists, therapists, nurses, and nutritionists. Interprofessional collaboration in managing older inpatients has been shown to reduce readmission rates, which can drive interprofessional education among the residents [11].

Participants

The research participants comprised general medicine trainees from the General Medicine Department of the Unnan City Hospital. Three medical teachers specialized in family medicine at the Unnan City Hospital during the study period. Before joining the Unnan City Hospital, the medical residents had received rural family medicine education at medical universities and tertiary hospitals. They had also trained in family medicine at rural hospitals for a year with medical teachers and other family medicine residents. One resident started training in the curriculum in 2018 and 2019 and three in 2020, 2021, and 2022. In 2022 and 2023, these 10 physicians (two family physicians and eight family medicine residents) were part of the General Medicine Department.

Educational content for integration and psychological safety

The department trains medical residents using an educational curriculum based on the Japanese Primary Care Association’s Board of Family Medicine, which was developed according to the World Standard of Education of Family Medicine. This curriculum can be used to simultaneously educate a maximum of three residents considering the educational capacity of the hospital. The hospital has a family medicine residency program with three teachers. Under this curriculum, residents experienced various clinical scenarios while treating their patients. In their first year, residents worked at the Unnan City Hospital and treated common diseases in both inpatient and outpatient settings. The next year, they worked at a rural clinic (Kakeya Clinic) for six months to learn about home care and community-oriented primary care. To broaden their scope of practice in internal medicine, pediatrics, and emergency medicine, the residents also worked at a general or community teaching hospital for 18 months. Each clinical setting included a medical teacher [12].

By clarifying each team member's role, we implemented the educational methods of near-peer learning. We adopted the shared-reading approach to improve psychological safety and the generation gap in our department as part of a process improvement program implementation. First- and second-year senior residents were responsible for patient management and educating medical students and junior residents. In addition, they were instructed to accompany students/junior residents to senior residents’ outpatient units and wards as much as possible and reflect on what students/junior residents had learned. Third-year residents were responsible for consulting and providing appropriate feedback to first- and second-year residents and for the overall management of senior residents. Third-year residents also liaised between supervising physicians and second-year residents. The medical teachers managed the entire residency while discussing patient management with the third-year residents [6,13].

The shared-reading approach used medical topics based on participants’ interests in medicine. Initially, the first researcher (RO) discussed the participants’ clinical questions and learning difficulties in the educational program to identify a topic to improve their learning. After selecting a topic, an appropriate book was chosen and shared with the interested participants. Each shared-reading group consisted of three to four members. The book chosen for shared reading was divided into several sections, and each group focused on one medical book at a time. Group members purchased their book copies and were encouraged to read a section each day. They shared their learning points with a closed social media group, where each member commented on each post based on their learning experiences [14].

The topics and books used in the shared reading were related to family medicine and chronic medical conditions, such as concepts of family medicine, cardiovascular disease, fever of unknown origins, general internal medicine management, and palliative care. Only Japanese texts were used because Japanese medical students tend to avoid reading English content [14]. The learning accumulated through shared reading was presented at the Department of Family Medicine conferences at the Unnan City Hospital, enabling members from all teams to expand their knowledge. Anyone interested in the content could join a shared-reading group anytime. Also, the participants were free to leave the group when they felt they did not have time to read. 

Data collection

Semi-structured Interviews

RO conducted semi-structured interviews to investigate the participants’ perspectives regarding transitioning from one educational setting to another in general medicine education. The interview guide included the following three questions: “What is the difficulty in transitioning from one educational setting to another in general medicine education?” “What are the benefits of transitioning from one educational setting to another in general medicine education?” “How would you consider revising the curriculum to mitigate the difficulty transitioning?” Each interview lasted approximately 30 minutes and was recorded and transcribed verbatim.

Data analysis

This study employed thematic analysis for data interpretation, explicitly following Braun and Clarke's (2006) six-phase methodology. This approach provides a structured process for identifying, analyzing, and interpreting meaningful patterns in the data [11]. Initially, the primary researcher (RO) immersed themselves in the data by repeatedly reviewing field notes and semi-structured interview transcripts, thereby forming preliminary insights. Systematic coding of the entire dataset ensued, generating initial codes pertinent to the research questions.

Subsequently, RO, in collaboration with a second researcher (CS), categorized these codes into potential themes, ensuring that all relevant coded data extracts were grouped under these themes [15]. This thematic framework underwent a meticulous review process, examining the coded extracts and the dataset. In instances of disagreement between researchers, themes were refined, which included splitting, combining, or discarding them as needed. Each theme was further honed to define its scope and focus, culminating in the development of distinct definitions and titles for each.

RO and CS engaged in continuous dialogue throughout this process to achieve consensus. The finalized themes were then documented by RO using descriptive and impactful examples, aligning the analysis with the research questions and relevant literature. For efficient data management, the study employed NVivo 11 software (QSR International, Melbourne, Australia).

Reflexivity

The researchers and participants co-created this study's results through interactions. The research team members possessed diverse expertise and perspectives on rural medical education. A family physician and medical teacher, RO graduated with a master’s degree in medical education, public health, and family medicine and has experience in practice, education, and qualitative research in rural settings. A medical educator and professor at a medical university, CS graduated from a medical university and specialized in community healthcare management and education. To prevent biases, the research team cautiously discussed the findings of individual data analyses. We explored alternative viewpoints while interpreting the data.

Ethical considerations

The participants’ anonymity was maintained throughout the study to ensure confidentiality. All participants provided written informed consent before participating in each shared-reading session. This study complied with the Declaration of Helsinki and subsequent amendments. The Unnan City Hospital Clinical Ethics Committee approved the study protocol (no. 20230026).

## Results

This study research provides insights into perceptions and experiences based on a thematic analysis of the general medicine education curriculum’s lateral integration between learning contents and contexts. The results are tabulated and summarized in Table 1.

**Table 1:** Themes and explanations

Theme	Explanation
Confusion due to the transition from acute to chronic clinical settings	Participants initially felt overwhelmed when transitioning from hospital medical care to clinic-based care, as the focus shifted from acute to chronic phases. They had a sense of not being able to keep up with the transition from acute to chronic care and were uncertain about how to proceed and how often they should see patients. With their limited clinical experience, the participants felt resistant to dedicating themselves solely to chronic phase medical care. Instead, they wished for an environment where they could gradually learn about the chronic phase while still being somewhat exposed to acute medical care
Monotony due to the loss of some clinical experience	Participants felt that moving from regional hospitals to clinics posed challenges in maintaining motivation because of the sudden absence of handling hospitalized patients and acute-phase responses. While attending a new educational facility initially propelled their learning due to the change in environment, after a certain period, the participants felt that their learning opportunities had diminished. They also felt limitations in their personal reflections
Disconnection between learning contexts	Participants, who conducted outpatient services daily, felt that given the remoteness of the location, it was challenging to know to what extent they could reliably follow up with the patients they treated. They were seeking feedback on a “just in case” basis. The participants felt a strong sense of unease about their diminishing connection with their primary training hospital. They believed that it would be even better if they could expand their clinical abilities based on what they had learned at the primary hospital
Anxiety as a community leader	Participants realized that within hospital medical care, there was a lack of perspective focused on the community. When transitioning to work in clinics, they felt the need for an educational approach that emphasized understanding the community perspective beforehand. The participants recognized a different type of leadership requirement when working in clinics. They felt uneasy about taking on a central role in managing clinic operations and sensed the need to advance their leadership learning. Given the scarcity of regional medical professionals, there was often only one doctor in the clinic, which increased the need for communication with other community healthcare professionals. The participants found that engaging in unprecedented levels of communication with various other professions made smooth interactions challenging because of a lack of understanding of the diversity and working styles of these professions

**Figure 1:**
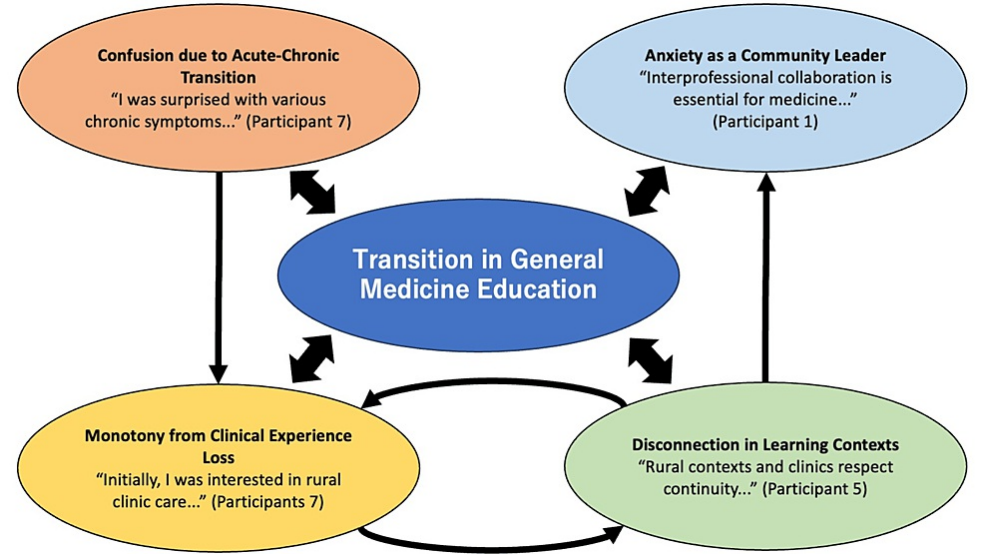
Theoretical model of transition in general medicine education settings

Figure 1 illustrates the theoretical model of transition in general medicine education settings.

Confusion due to the transition from acute to chronic clinical settings

Participants transitioning from hospital medical care to clinic-based care faced challenges as the focus shifted from acute to chronic phases. The rapid shift left them feeling overwhelmed and struggling to adjust to the demands of chronic patient care. Participant 7 stated, “I was surprised by one patient's chronic symptoms. I had only one medical problem to solve in the hospital, mainly during discharge. However, I must deal mainly with chronic diseases in the clinic by reviewing the previous medical history of patients.” 

Given their limited experience, the participants hesitated to immerse themselves entirely in chronic phase medical care. Based on their experiences with various chronic care needs in the clinic, the participants realized their lack of preparation for chronic care in community medicine. They reported that they could not listen to previous patients’ chronic symptoms. Participant 1 stated, “I did not imagine there would be such a wide variety of chronic symptoms. Patients could not describe such symptoms to me during hospital care because of acute symptoms. I should have considered their chronic phases during the care.” 

A prominent desire emerged among participants for a gradual introduction to the chronic phase without hindering their exposure to acute medical care. By reflecting on their previous hospital experiences, the participants realized that they could have various experiences, enabling them to learn about acute and chronic symptoms. They were motivated to learn more about chronic symptoms, suggesting effective continuity of learning from hospitals to clinics. Participant 2 stated, “I had various cases through which I could learn about chronic symptoms, but I did not understand the importance of those symptoms at that time and I could not identify them, the medical teachers should encourage us to learn more about chronic diseases in the hospital.” 

Monotony due to the loss of some clinical experience

The move from regional hospitals to clinics led to challenges in terms of sustaining participant motivation, which was attributed to a lack of interaction with hospitalized patients and acute-phase medical conditions. Participant 3 stated, “In a rural clinic, there are many things to learn and get feedback from my teachers. However, I felt isolated in the rural clinic because I treated the patients without supervision. In addition, the feedback frequency was reduced because of the diminished communications with medical teachers.” 

Although the new educational setting initially appeared beneficial, the perceived learning opportunities eventually dwindled. The participants also recognized limitations in reflecting on their personal clinical experiences. Moreover, some participants lost interest in the chronic care of older patients in rural clinics. Participant 7 stated, “Initially, I was interested in rural clinic care as it was a new experience. However, everyday chronic care did not stimulate my learning, and I did not learn anything new in the last month of working in rural clinics and chronic care.” 

Disconnection between learning contexts

The participants, particularly those in remote settings, expressed difficulty in tracking the progress of patients they had personally treated. In the absence of regular feedback, they often relied on a “just in case” approach. The participants hoped to receive regular follow-ups from medical teachers for education on continuity of care in rural clinics and to improve the care of patients in rural clinics. Participant 5 stated, “Rural contexts and clinics respect continuity of care as primary care facilities more than hospitals. Patients in rural clinics hope for continuity. I hoped to be followed up by medical teachers more frequently to ensure continuity.” 

The perceived waning connection with their primary training institutions was a significant concern. Participants believed that their clinical capabilities would be enhanced if they could further integrate the skills and knowledge acquired from their primary training hospitals. The participants insisted that the integration between community hospitals and rural clinics regarding family medicine education should be enhanced more comprehensively and facilitate the medical learners to learn various contextual learning methods. Participant 3 stated, “Educational environments and learning contents should be better connected and integrated. I hope that medical teachers will facilitate medical learners to prepare to learn different contexts with acquired competency from one setting to another“.

Anxiety as a community leader

This study highlighted a renewed realization among participants regarding the missing community perspective in traditional hospital medical care. Transitioning to clinic-based roles underlined the need for an educational framework that prioritized the community’s perspective. The participants insisted that they needed to learn more about community care while working at rural clinics to improve the care of older people. Participant 2 stated, “I learned about community care previously for working in rural clinics. However, the reality differed, as the interaction with rural citizens demanded a different understanding of communities. Medical educators should facilitate the learning about communities.”

The participants also identified the importance of leadership roles in clinics, which was accompanied by feelings of anxiety about clinic administration. With often just a single physician available in rural clinics, the need for efficient communication with other community healthcare professionals became evident. Increased communication with diverse professions presented several challenges, primarily due to unfamiliarity with these professionals’ roles and working methodologies. Participant 1 stated, “Interprofessional collaboration is essential for medicine. I am used to such collaborations in hospitals. However, rural communities have various healthcare professionals and community workers who support citizens’ lives. As a rural healthcare leader, I should have learned more about them to ensure effective care of patients in their communities.” As other participants stated, family medicine education is needed to improve the quality of education in medicine as well as leadership in teams and communities.

## Discussion

The thematic analysis of the general medicine education curriculum’s lateral integration offers profound insights into the perceptions and experiences of medical participants, primarily when transitioning from hospital settings to clinics. These findings raise some critical pedagogical questions and provide valuable suggestions for curriculum revisions.

Confusion due to the transition from acute to chronic settings should be mitigated through the simulation of chronic care in hospital settings and the provision of acute care practice while working in clinics. Participants frequently highlighted the dissonance between hospital- and clinic-based care, especially in the shift from acute to chronic patient management. This suggests that the education curriculum could benefit from a more seamless transition, bridging the gap between these settings [16]. Previous studies have shown that discrepancies between learning contexts and content can demotivate learners in educational curricula [17,18]. Training modules that simulate chronic care scenarios in hospital settings or introduce chronic management principles during hospital rotations can provide residents with the tools and confidence they need to excel in clinic-based care [19]. Educational curricula in general medicine should set educational contexts, including acute and chronic care while teaching chronic care.

For medical trainees, learning chronic care can be a monotonous experience as the clinical experience in primary care clinics is less stimulating. As this article shows, some participants expressed a loss of motivation, and the feeling of isolation underscores the need for continuous mentorship, even in remote clinical settings. Learning general medicine in community hospitals should drive the connection between acute and chronic care [20]. Furthermore, introducing diverse clinical cases and simulations can break the monotony and keep learners engaged [21]. Collaborative approaches, where learners can periodically reconnect with their hospital peers and mentors, can also be explored [22]. Medical educators should facilitate medical learners to reflect on their experiences in chronic care with their peers, thereby mitigating their monotonous learning.

Disconnection between learning contexts should be mitigated through continual reflection among medical teachers and learners. As this research shows, the participants’ concerns about the waning connection with their primary training institutions underline the importance of continuity in the learning experience. It is necessary to ensure that learners maintain ties to their primary institutions, enabling them to draw on previously acquired skills and knowledge [12,23]. Previous studies have shown that constant communication and reflection between medical teachers and learners improve their relationships in educational contexts and psychological safety in rural settings [24,25]. In rural contexts, teacher-learner relationships can be facilitated through regular virtual check-ins, case discussions, or integrating periods of returning to the primary institution as shared reading and continual reflection [14]. Also, in rural contexts, few healthcare institutions can provide medical education. Therefore, rural communities can get a few medical institutions to focus on educational functions and increase stakeholder interactions, thereby improving the quality of education.

Anxiety as a community leader among medical learners can be mitigated by gradual exposure to community activities and facilitation by medical educators. The study findings emphasize the importance of leadership training in medical education, particularly for those working in under-resourced settings where interprofessional collaboration and health dialogue with citizens are crucial [26]. In medical education, gradual exposure to new learning content with feedback from medical teachers is essential [27]. Medical curricula can integrate gradual exposure to community medicine with leadership courses, emphasizing communication, team management, and community engagement through collaboration with medical teachers and community stakeholders [28]. Considering the distinctive challenges faced in rural settings, a specialized module focusing on rural healthcare leadership, encompassing the diverse spectrum of healthcare professionals and community stakeholders, can be beneficial [28]. Furthermore, the participants’ realization that they lack a community perspective during their traditional hospital-based training underscores the need to infuse community medicine principles and practices throughout the medical curriculum. While the curriculum may already address these issues in dedicated segments, these findings suggest the value of a more integrative approach.

**Figure 2:**
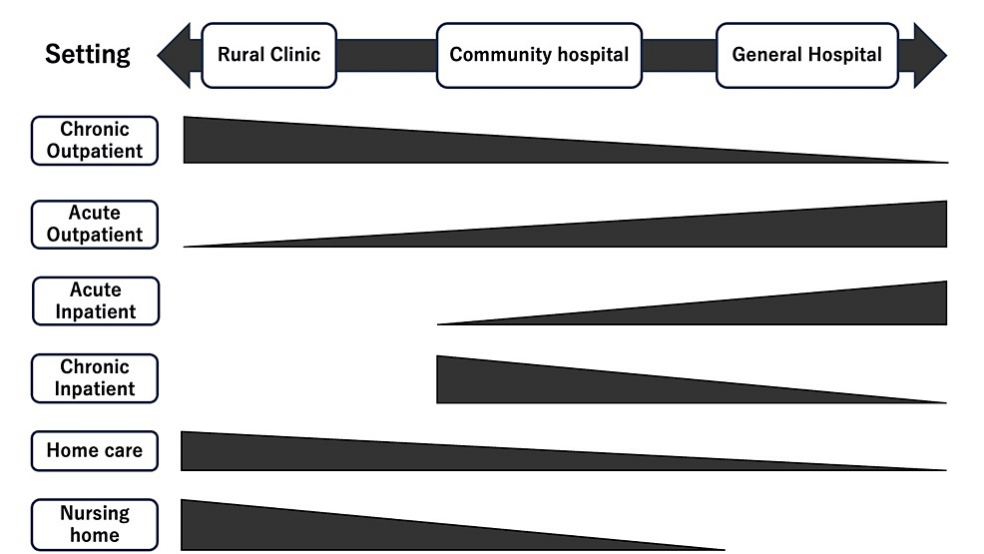
Range of scope of various educational settings

One solution for maintaining the continuity of general medicine education is the involvement of community hospitals in educational curricula in rural contexts. This research shows that the need for more medical educators and the gap in experiences between general hospitals and clinics can place constraints on general medicine education in rural settings. The demand for educational contexts endowed with the learning contents of both hospitals and clinics is high [29]. Community hospitals in rural communities can be an ideal learning atmosphere for general physicians (Figure 2).

Community hospitals provide inpatient and outpatient care in rural contexts, although patients’ disease severity and multimorbidity can differ from those in general hospitals. Community hospitals can deal with providing inpatient care at home and palliative care with a few medical staff including nursing homes, enabling medical learners to learn about communities and administration instead of working and learning at rural clinics [30]. Community hospitals should be included in curricula for practical learning in rural general medicine education.

This study has a few limitations that need to be acknowledged. Since the participants in this study may not represent the broader population of medical students or professionals undergoing similar transitions, the results may only apply to some medical education settings or geographical regions. Thematic analysis, being qualitative in approach, relies on the interpretation of the researchers, which may introduce an element of subjectivity. Despite rigorous analysis, missing nuances or overemphasis on themes is still possible. Participants’ reflections on their experiences might be influenced by recall bias, where certain events or feelings might either be exaggerated or minimized based on the time elapsed since the event. Moreover, while offering significant depth, the study’s qualitative approach does not provide quantitative metrics that can measure the extent or magnitude of the identified issues. Cultural or regional specifics may influence the experiences shared by the participants. Therefore, the findings might be limited in their applicability to regions or cultures different from the study’s context. The voluntary nature of participation might have led to a selection bias, with individuals with powerful feelings or experiences being more likely to participate.

## Conclusions

The thematic insights from this study highlight a pressing need for reform in the general medicine education curriculum. Comprehensive integrative modules, mentorship programs, and leadership training are essential to achieve this. These additions can ensure that medical professionals are thoroughly equipped to tackle the diverse and evolving challenges in various healthcare environments. This approach could significantly enhance the quality of medical education, directly impacting future healthcare professionals' effectiveness and adaptability.
